# Evolutionary Patterns of Non-Coding RNA in Cardiovascular Biology

**DOI:** 10.3390/ncrna5010015

**Published:** 2019-01-31

**Authors:** Shrey Gandhi, Frank Ruehle, Monika Stoll

**Affiliations:** 1 Institute of Human Genetics, Department of Genetic Epidemiology, University of Münster, 48149 Münster, Germany; shreygandhi1990@uni-muenster.de (S.G.); ruehle@uni-muenster.de (F.R.); 2 Genetic Epidemiology and Statistical Genetics, Department of Biochemistry, CARIM School for Cardiovascular Diseases, 6200 MD Maastricht, The Netherlands; 3 Maastricht Center for Systems Biology (MaCSBio), Maastricht University, 6200 MD Maastricht, The Netherlands

**Keywords:** non-coding RNA, cardiovascular disease, evolutionary conservation, lncRNA, circular RNA, miRNA

## Abstract

Cardiovascular diseases (CVDs) affect the heart and the vascular system with a high prevalence and place a huge burden on society as well as the healthcare system. These complex diseases are often the result of multiple genetic and environmental risk factors and pose a great challenge to understanding their etiology and consequences. With the advent of next generation sequencing, many non-coding RNA transcripts, especially long non-coding RNAs (lncRNAs), have been linked to the pathogenesis of CVD. Despite increasing evidence, the proper functional characterization of most of these molecules is still lacking. The exploration of conservation of sequences across related species has been used to functionally annotate protein coding genes. In contrast, the rapid evolutionary turnover and weak sequence conservation of lncRNAs make it difficult to characterize functional homologs for these sequences. Recent studies have tried to explore other dimensions of interspecies conservation to elucidate the functional role of these novel transcripts. In this review, we summarize various methodologies adopted to explore the evolutionary conservation of cardiovascular non-coding RNAs at sequence, secondary structure, syntenic, and expression level.

## 1. Introduction

Myocardial and vascular ailments are complex systemic diseases, successively leading to chronic cardiac complications. These cardiovascular diseases (CVDs) encompass a broad range of disorders including atherosclerosis, inflammatory heart disease, arrhythmias, and congenital heart disease among others. Cardiovascular disease remains the major cause of death in the world, exceeding deaths due to communicable diseases such as malaria, HIV/AIDS, and tuberculosis [1,2]. In 2016, an estimated 17.9 million people died from CVDs all across the world [1,3]. Approximately 85% of deaths in these cases are due to myocardial infarction and stroke. Currently, 80% of CVD mortality occurs in developing nations and is expected to be the major cause of mortality in most developing nations by 2020. In 2011, three in every 10 deaths were caused by CVD and it is estimated that by 2030, 23.3 million people will die annually due to CVD [1,2].

In addition to sex, age, and other environmental factors, genetic factors are major drivers for complex cardiovascular diseases [2,3]. Over the past years, several genetic studies have tried to correlate genotype with phenotype, i.e., to identify gene–gene and gene–environment interactions. Genome-wide association studies (GWAS) compare the frequencies of genetic variation mapped as e.g., single nucleotide polymorphisms (SNPs) in individuals with a given disease and in control individuals from the same population or ethnic background. The genetic variants predisposing to CVD range from deleterious mutations responsible for Mendelian diseases to common polymorphisms that contribute to disease risk with a modest effect at the individual level. Notably, in most complex CVDs, common variants are not sufficient to explain the entire disease risk and a large part of genetic variance remains unaccounted for [4]. This “missing heritability” can be explained in part by rare genetic variants, which in sum can have large effects.

Most of the CVDs show complex inheritance patterns due to the complex interactions between several genes and non-genetic factors. The current environment and changing dietary patterns followed by majority of the world population add to the genetic susceptibility to culminate as deadly complex cardio-metabolic complications [2]. Other factors such as sleep patterns, climate, and physical inactivity also contribute to the pathophysiology of these diseases. High blood pressure, hypercholesterolemia, obesity, diabetes, smoking, alcohol consumption, etc. are other pathological accelerators that increase the risk of developing CVD and are often found to be strongly associated with CVD linked mortality [2].

Over the past decade, several novel non-coding RNAs (ncRNAs) have been discovered with importance in cardiovascular biology. This has been accelerated by next generation sequencing (NGS) methods which have been applied to cardiovascular genomics, transcriptomics, and epigenomics to explore the correlation between the genotype and complex cardiac phenotypes [5]. Such leads not only enhance the understanding of disease pathogenesis, but they also identify non-coding transcripts that can be quantitatively assessed as novel biomarkers [6].

Apart from mutations in coding genes such as *APOE* [7], *PAI-1* [8], *ACE* [9], and *MTHFR* [10], numerous signals have been detected in the non-coding genome. In fact, there is an increasing body of evidence that suggests the presence of the vast majority of associated variants in non-coding regions. This observation, accompanied by the dysregulation of various non-coding transcripts in CVD patients, has shifted the focus towards understanding ncRNA biology. The number of these transcripts has steadily grown over the years, yet the biological roles of most of them are largely unknown. This discrepancy is due to the lack of proper technologies to probe transcript functions at a genomic scale.

One approach to tackle this problem has been the use of comparative genomics to identify homologous sequences for the gene of interest in other organisms. The conservation of sequences under selection pressure hints at an enduring functionality similar to the ancestral ortholog. However, unlike protein coding transcripts, there are serious challenges when dealing with some classes of ncRNA transcripts, especially long non-coding RNAs (lncRNA). The lack of consensus sequence similarity and rapid evolutionary turnover makes the identification of orthologous sequences very challenging. Recent developments in the field have tried to address this issue by looking at other dimensions of lncRNA conservation including structure, synteny, and spatio-temporal expression patterns.

## 2. Heart and Non-Coding RNAs

### 2.1. Long Non-Coding RNAs

While a large proportion of the human genome is known to be transcribed, only ~2% of the genome appears to code for proteins [11,12]. Recent technological advances in sequencing combined with an improvement in computational algorithms has enabled us to study the complex nature of transcriptomes. These advancements have led to improved characterization of non-coding RNA molecules and established them as important regulators of cellular and tissue functions. These ncRNAs can be classified into two major groups based on the length of the transcripts. The small non-coding RNAs, which are shorter than 200 nucleotides and other being long non-coding RNAs.

Long non-coding RNAs play an important role in the development of specific tissues of the human body. They have regulatory functions in maintaining the cellular morphology and differentiation, acting via both cis and trans interactions [13]. They act, for example, as molecular sponges for microRNAs (miRNAs) and RNA-binding proteins (RBPs) to inhibit or enhance the expression of the genes. Few studies have shown that lncRNAs also participate in molecular signaling by being transcribed at specific spatial and temporal points [14], acting as the bridge between coding and non-coding biology via formation of RNA–DNA–protein complexes. The cellular localization of lncRNAs is important in determining their functional properties [15]. While most of the lncRNAs are enriched in the cytoplasm or ribosomal fractions, some exclusively reside in the nucleus [16]. Long non-coding RNAs, which mostly reside in the nucleus, have been shown to regulate gene expression in cis or trans by the formation of RNA–DNA complexes and recruiting chromatin modifiers or transcription factors. Long non-coding RNAs, which are exported to the cytoplasm, play important roles in modulating translation, acting as competing endogenous RNA, and regulating protein modifications among others. The association of lncRNAs with ribosomes has been linked to its role in regulating translation, degradation, and formation of short peptides [16,17,18].

Long non-coding RNAs have been identified as key regulators of gene regulation in the development and function of the cardiovascular system. Cardiomyocyte differentiation and development, heart wall development, cardiac morphology, cardiac cell depolarization, and repolarization are some of the core functions that are affected by the lncRNA machinery in the human heart [19,20,21,22]. Several research studies identified the lncRNAs acting as miRNA sponges to affect the vascular remodeling, cardiomyocyte dysfunction, hypertrophy, and phenotypic switch of vascular smooth muscle cells from a contractile to a synthetic state in case of ailing heart [23].

Many annotated and novel putative lncRNAs have a heart specific expression pattern and multiple examples of divergently expressed lncRNA–mRNA pairs have been identified, suggesting functional relationships. With major roles to play, lncRNAs have been identified as mediators of maintenance of cardiovascular health with many lncRNAs as potential biomarkers (Table 1).

### 2.2. MicroRNAs

Another major class of non-coding RNAs are the small non-coding RNAs, with miRNAs being most notable. MicroRNAs are ~22 nucleotides single-stranded molecules which primarily function as post-transcriptional regulators of gene regulation. They are the functional unit of the RNA-induced silencing complex (RISC), which bind to its target mRNA in a sequence-dependent manner resulting in the degradation or deadenylation of the mRNA [49]. Additionally, miRNAs can also be sequestered by other lncRNA or pseudogenes introducing a new layer of regulatory complexity [50].

MicroRNAs play an integral part in all the facets of cardiovascular biology, including smooth muscle maturation and proliferation, endothelial function, and regulation of genes involved in cardiogenesis. Several pathological conditions, such as atherosclerosis, heart failure, cardiomyopathy, and myocardial fibrosis are shown to result from the dysregulation of miRNA (Table 2).

A large effort has been placed in developing miRNA mimics and anti-miRNA inhibitor molecules as therapeutic interventions to regulate disease physiologies. One of the first miRNA-dependent therapies was developed in 2008, where a highly specific antagomir of miR-21 was developed for the attenuation of cardiac dysfunction in rodent model of cardiac fibrosis [51]. The high stability of circulating miRNAs in plasma and their differential expression in disease phenotypes also makes them excellent candidates as biomarkers in CVD.

### 2.3. Circular RNAs

Recent development in RNA sequencing technology has facilitated the characterization of several novel RNA transcripts. Circular RNAs (CircRNAs) represent one such emerging class, which has been identified across multiple species including archaea, fungi, plants, fish, insects and mammals [65,66,67]. These transcripts have been shown to perform a myriad of regulatory roles in multiple biological processes. Circular RNAs are known to function as miRNA sponges [66,68], splicing competitors [69], protein binding/sequesters [70], and transcription [71] and translation [70] regulators of the host gene. Some circRNAs have also been shown to produce proteins using the translational machinery in a cap-independent manner [72,73]. The expression of circRNAs is spatio-temporally regulated and plays a critical role in the development and pathogenesis of several diseases including cancer, neurological and CVD [74,75,76].

Many transcriptomic studies have focused on the identification of circRNAs [77,78] during cardiac development and pathological conditions. Interestingly, most of these studies detect differential expression of multiple circRNA isoforms specifically from *TTN* and *RYR2* genes. These are known genes which play an important role in cardiovascular biology, yet the functional characterization of their circular isoforms remains to be established. Recent studies have tried to interpret the role of many candidate circRNAs in cardiovascular development and disease, which is summarized in Table 3.

The efforts to identify circRNAs is also in part due to the promise, which these novel transcripts offer as potential biomarkers. For one reason they are expressed in a cell-specific manner. Another reason is the lack of free ends which renders them resistant to exonuclease-mediated degradation. CircRNAs have been shown to have a median half-life of at least 2.5 times higher than their linear counterparts [79]. Apart from being highly stable, they have been detected to be circulating in the blood and are present in plasma as well as extracellular vesicles [80,81,82]. One study also showed their presence in cell-free saliva which makes them excellent candidates for non-invasive detection [83]. Wesselhoeft et al. engineered circular RNAs for the production of proteins and showed their prowess as robust and stable protein producers. This also suggests their potential as therapeutic vehicles [84].

## 3. RNA-Sequencing for Identification of Non-Coding RNA

RNA sequencing (RNA-seq) has emerged as one of the major facilitators for the identification and characterization of ncRNAs. RNA sequencing characterizes CVD by studying transcriptome-wide expression profiles, alternative splicing patterns, and regulatory networks that provide deeper information of the biochemical pathways altered in the diseased condition and possible modifiable genome level interactions. RNA sequencing has enabled us to compare gene expression in diseased and non-diseased tissues or blood components to yield a set of genes that might explain the pathological condition. Several variants of RNA-Seq protocol have been developed to study spatio-temporal complexity of individual components of the transcriptomes. These protocols have been accompanied by computational methodologies to assist in the proper quantification of transcripts [91].

Most of the lncRNA detection studies either involve poly-A enrichment or rRNA depletion before library preparation. While mRNAs and many lncRNAs contain a poly-A tail, these molecules can be detected using poly-A enrichment. However, since there are many non-polyadenylated lncRNAs, these transcripts will not be captured. Sequencing protocols involving rRNA depletion enable us to cover the whole diversity of transcripts. Thus, the choice of sequencing methodology highly depends on the desired targets to be sequenced and economic viability. Multiple algorithms have been developed to help distinguish between protein-coding and lncRNA transcripts [92]. Small RNA-seq enables the identification of miRNA and other small RNA species using size selection techniques. Although, total RNA-seq can capture circRNA transcripts, specialized protocols have been developed to enrich for circular transcripts by selecting against poly-A transcripts. Several algorithms have also been developed to facilitate the identification of back-splicing junctions, which are a hallmark of circRNA transcripts [93,94]. Recently, the focus has also shifted towards the quantification as well as the relative abundance of these molecules compared to linear counterparts of the host gene [95,96].

## 4. Experimental Methodologies to Explore ncRNA Functionality

Despite the growth in the number of lncRNAs and database resources, most of the lncRNAs remain uncharacterized [97]. While miRNA function and their binding targets are better understood, these resources remain far from completion [98]. Many resources provide information about experimentally validated functions of lncRNAs, yet just looking at the number of represented lncRNAs makes the void quite evident [99,100].

One way of addressing this gap is to search for the homologous transcripts in related organisms. The main assumption behind most of the ortholog identification studies is that they also share biological function. However, due to limited consensus in the methods for identification of ncRNA homologs, this may not always be true. Therefore, apart from verifying the presence of individual lncRNAs, there is also an urgent need to experimentally validate their biological role.

Despite recent progress, functional genomics is yet to be completely exploited to understand the lncRNAs’ functions, their interactions, as well as mechanism of regulation and physiological relevance. In recent years, novel methodologies have been developed to probe the function of individual transcripts mostly involving its overexpression, knockout or knockdown studies (Table 4) [101,102]. Several high-throughput techniques also enable the investigation of interactions of lncRNAs with DNA, RNA, and proteins (Table 4) [103]. As lncRNAs are pivotal to cardiovascular biology, functional validation can help deepen our understanding of their biological implication in development and disease.

## 5. Conserved Nature of Non-Coding RNAs

The evolutionary conservation of ncRNA has been a topic of intense research in the last few years. While some classes of ncRNA such as miRNAs are considered highly conserved, establishing the conservation of lncRNAs remains challenging. Most of the earlier efforts were focused on establishing these orthologous relations based on sequence conservation. Some studies tried to identify segments of the genome which were ultra-conserved across species and found that majority of them were located in introns and intergenic regions [104]. Further studies confirmed that most of these regions are indeed transcribed into lncRNA sequences [105].

**Figure 1:**
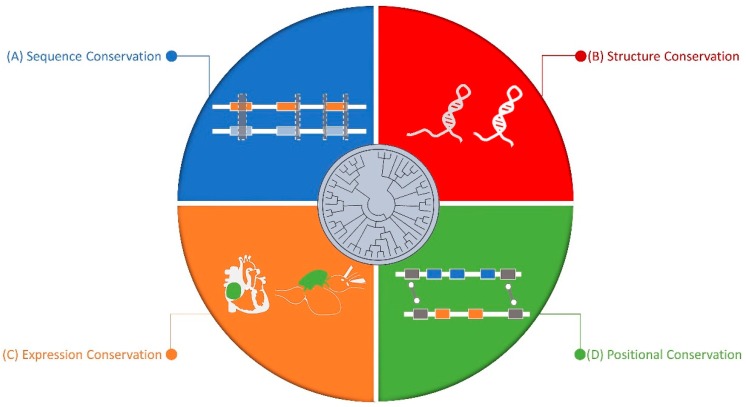
Dimensions of long non-coding RNA (lncRNA) conservation: (**A**) Sequence Conservation: sequence homology or/and conserved gene structures across different organisms. (**B**) Structure Conservation: lncRNAs can form conserved secondary or tertiary structure. (**C**) Expression Conservation: the expression patterns of lncRNA can be spatio-temporally conserved across species. (**D**) Positional Conservation: the syntenic location of lncRNA with respect to its neighboring genes is conserved across species.

On the other end of this spectrum, Pollard et al. [106] investigated regions within humans with high sequence diversity but were conserved in other species, and also found them to be mostly within non-coding regions. They argued that the lack of sequence conservation does not mean lack of function. Although, sequence conservation still remains the primary method for identifying orthologs, many researchers have tried to complement this with structure, synteny, and expression level conservation (Figure 1).

### 5.1. Sequence Level

The precise detection of homologous transcripts has mainly relied on sequence level conservation between species. Over the years, several resources have been developed to infer these orthologous relations which can be divided into tree-based or graph-based algorithms [107]. The main principle behind these methodologies is to differentiate between orthologs, which are a result of speciation events and have the same function and paralogs resulting from gene duplication and can differ functionally. Several attempts have also been made to compare and standardize the methodologies in order to get a more accurate ortholog detection [108,109,110]. However, the fact that lncRNAs are not well conserved at the sequence level has limited their application beyond coding genes. In fact, due to the degree at which the sequences have diverged, it is sometimes impossible to call any ortholog. There are only a handful of known lncRNAs which show sequence conservation similar to coding genes [111].

With decreasing sequencing costs, it has become feasible to investigate genome-wide lncRNA sequences across organisms. Some studies which have attempted to look at genome-wide sequence homology in lncRNAs, mainly employ a reciprocal best hit method involving two-way sequence alignment (Table 5). Most of these studies looked at transcriptome patterns across different organs to capture complete transcriptomic heterogeneity across each species [112,113]. Recent attempts have tried to improve this approach by utilizing synteny and structure-based methods to aid in the identification of orthologs [114]. Nonetheless, these studies mostly agree that lncRNAs undergo rapid evolutionary changes and the sequences are rarely conserved beyond a particular evolutionary point.

However, the transcriptome profiles evolve more dynamically and in many cases the comparison of transcribed sequences may not provide the complete perspective. The changes in splicing patterns and exonic boundaries result in lncRNAs of one species aligning to non-transcribed regions in the other. Moreover, in some cases there is no sequence similarity between organisms except near the 5′ end and promoter sequence of the lncRNA. In fact, some studies have also pointed out that the promoter regions of lncRNAs are often as conserved as the promoters of protein coding genes [113,117]. These complexities make it crucial to correctly identify and characterize lncRNA orthologs. Numerous lncRNAs, such as MALAT1, HOTAIR, GAS5, CARMEN and CHAST, have been identified in humans with some degree of sequence conservation across other organisms. Still there are many other lncRNAs in CVD, whose orthologs are yet to be identified.

### 5.2. Structure Level

Non-coding RNAs, especially miRNAs are known to form secondary structures, which are important for its interactions with other biomolecules and thus their function. Just like other mRNA transcripts, lncRNAs are also known to form stable secondary structures [118]. The absence of significant sequence conservation does not mean lack of selection at the structural level [119]. This is evident in case of the telomerase RNA and the stem region of miRNAs, which even in the absence of sequence similarity maintain structural integrity. However, this hypothesis has been tested with limited success in case of lncRNAs.

The fact that even random RNA sequences can form stable structures, suggests that it is not a necessary condition for a functional correlation at the sequence level. Indeed, there are contradictory views about the conservation of lncRNA secondary structures. While some studies suggest lack of any statistically significant conserved RNA structure for some lncRNAs, others have shown conserved structural domains in several lncRNAs [120,121,122,123]. In fact, several important cardiovascular lncRNAs, such as GAS5 and HOTAIR, have been shown to have some degree of structural conservation [124,125].

Over the last two decades, several studies have tried to use computational methods to look at the genome-wide RNA secondary structure conservation (Table 6). Most of these tools are based on sequence alignment, and thus require a certain degree of sequence conservation. Others have tried to overcome this obstacle by using conserved synteny as the basis for the identification of stretches for structural survey. However, most of these computational methods, irrespective of their intrinsic principle, suffer from low detection accuracy and their predictions rarely agree [126].

Recent advancements in high-throughput technologies have made it possible to probe RNA structures across the genome [139]. Methodologies such as PARS (parallel analysis of RNA structure), Frag-Seq (fragmentation-sequencing), SHAPE-Seq, DMS-Seq, among others, have been developed to determine RNA structures on a genome-wide scale. These experimental methodologies coupled with computational algorithms can greatly improve the accuracy of RNA structure prediction, thus improving our understanding of lncRNA structure conservation.

### 5.3. Synteny Level

Non-coding RNAs are known to regulate the expression of protein coding genes both via cis and trans-acting mechanisms. Although, most lncRNAs undergo rapid evolutionary turnover in terms of sequence and transcription, yet the syntenic relationship with neighboring genes appears to be preserved [114,140,141]. Many times, such lncRNAs display only local levels of sequence conservation mostly near the promoter region, which suggests that the transcriptional event from that loci is essential and the lncRNA itself might be of less importance. FENDRR and PVT1 are two lncRNAs, which are essential to cardiovascular biology and do not show high levels of sequence conservation, yet their relative location is conserved [114].

The fact that lncRNAs maintain their positional integrity across species, provides insight into the origins of these transcripts. Hezroni and co-workers [142] suggest some lncRNAs might be relics of ancestral genes which lost their coding potential. Ning et al. [143] suggested that many of the lncRNA-coding gene overlap pairs were a result of overprinting and not due to genomic rearrangements. Other recent findings suggest lncRNAs to be intermediaries leading to the origin of novel protein coding genes [144,145]. Chen et al. [141] also investigated the positional conservation of lncRNAs with respect to miRNAs, snoRNAs, and protein coding transcripts and suggested their classification based on evolutionary history.

This evidence suggests that the position of lncRNAs is important for the cis regulatory function. Long non-coding RNAs that are antisense to protein coding genes have been shown to influence nearly every aspect of gene expression regulation by interacting with DNA, RNA, and proteins of the respective coding gene [146]. In particular, lncRNAs overlapping protein coding genes display a high level of co-expression and tissue specificity resulting in their evolutionary retention [143]. Amaral et al. [147] described lncRNAs, which bear positionally conserved promoters in humans and mice, and were enriched at topologically associating domain (TAD) boundaries. Their findings indicated their role in the regulation of expression in neighboring genes and modulation of chromatin looping. These studies emphasize the importance of syntenic conservation on the functional properties of lncRNA.

### 5.4. Expression Level

**Figure 2:**
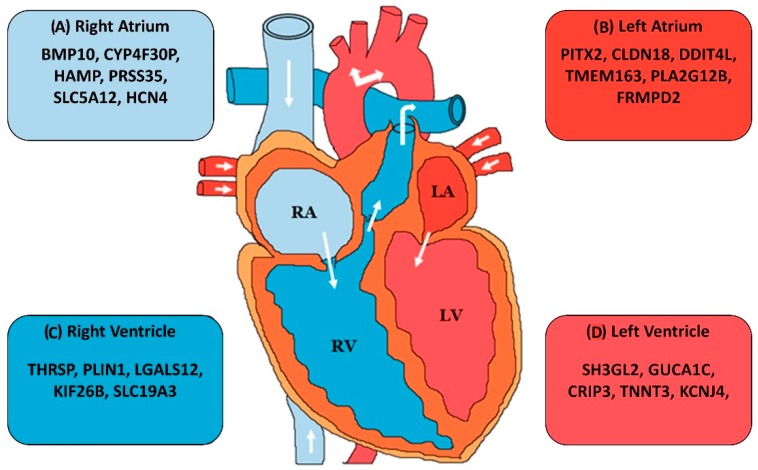
Chamber specific gene expression in human heart: the figure provides an overview of the genes which have been found to be enriched in the (**A**) right atrium, (**B**) left atrium, (**C**) right ventricle, and (**D**) left ventricle as compared to other heart chambers [150,151,152,153].

Transcriptome profiles of individual organs have been demonstrated to be more conserved across species than they are across organs within the same species [148]. Long non-coding RNAs are expressed at lower levels than mRNAs and less conserved at the sequence level, but they are known to be highly tissue specific [113,114]. It therefore becomes imperative to carefully match homologous tissues across species in order to capture the complete expression profiles. This specificity has also been observed for the human heart. Not only the transcriptome profile of the heart is different from other organs, recent studies have also demonstrated it to be different across the heart chambers [149,150]. These differences shed some light into the function and pathophysiology of heart related ailments. Indeed, there are known examples of non-coding transcripts which are expressed exclusively in a particular heart chamber (Figure 2), yet not much is known about this specificity. Future studies will provide deeper insight into the conservation of expression profiles across heart chambers and other tissue subtypes.

## 6. Conclusions

The advancements in NGS technologies have accelerated the identification of various novel ncRNA transcripts in CVD. However, only a handful of these transcripts have been functionally characterized. The lack of high-throughput experimental approaches to elucidate the role of these transcripts makes their functional investigation very challenging. The availability of well characterized genomes has led to the emergence of comparative genomics methodologies to functionally annotate them. These methods are highly dependent on sequence conservation across species, and thus, limited mainly to protein coding genes.

Although several lncRNAs show sequence conservation, the rapid evolutionary turnover has resulted in sequence divergence beyond recognition. Despite this, most of the lncRNAs appear to have conserved expression patterns and functions. Over the past years, several experimental methodologies have been developed to explore the structural elements within lncRNAs. These protocols have enabled us to investigate the genome-wide structural conservation of lncRNAs. Apart from this, many studies have tried to exploit the syntenic conservation of lncRNAs to improve the characterization of their homologs. These studies highlight the fact that there are several dimensions to interspecies conservation, and a lack of sequence conservation does not necessitate lack of function.

Novel and innovative approaches accompanied by improved experimental methodologies should aid to understand the functional implications of non-coding transcripts. In summary, future studies encompassing these dimensions of non-coding RNA conservation pose an exciting opportunity to investigate the role of non-coding RNAs in the cardiovascular system.

## Figures and Tables

**Table 1:** List of clinically relevant long non-coding RNAs (lncRNA) in cardiovascular biology.

Transcript	Host Gene	Organism Studied	Mechanism	Disease	Reference
Anril	*CDKN2B-AS1*	Human	Binds to *CBX7*, recruits *PRC-1* and *PRC-2* to *INK4* locus-leading to the repression of p15 and p16 transcription	Genetic risk factor for coronary artery disease (CAD) and myocardial infarction (MI)	[24]
BVHT	*BVHT*	Mouse	Activation of mesoderm posterior 1 (*MesP1*) and interacts with *SUZ12*, a component of *PRC2*, during cardiomyocyte differentiation	Impairs cardiomyocyte differentiation	[25]
FENDRR	*FENDRR*	Human, Mouse, Rat	Binds to the histone-remodeling *PRC2* complex and *TrxG*/*MLL* to modulate chromatin status	Low expression leads to cardiac hypoplasia	[26]
NOVLNC6	Intergenic	Mouse	Modulates expression of *MKX2.5*	Downregulated in dilated cardiomyopathy (DCM)	[27]
CARMEN	*CARMN*	Human, Mouse, Rat	Interacts with *SUZ12* and EZH2 of *PRC2* complex	Plays a critical role in maintaining a differentiated cardiac fate in mature cardiomyocytes in case of DCM and aortic stenosis (AOS)	[28]
KCNQ1OT1	*KCNQ1OT1*	Human, Mouse	Kcnq1 imprinted domain in heart development	Defects in KCNQ1 leads to cardiac arrhythmias, predicts left ventricular dysfunction	[29]
SENCR	*SENCR*	Human	Inhibitor of smooth muscle cell migration	Downregulated in CAD and MI	[30]
MALAT1	*MALAT1*	Human, Mouse	-	Involvement in the pathogenesis of diabetic cardiomyopathy	[31]
H19	*H19*	Human	Acts by targeting *VDAC1*	Regulates cardiomyocyte apoptosis in diabetic cardiomyopathy	[32]
RNCR3	*RNCR3/LINC00599*	Human, Mouse	miR-185-5p sponge	RNCR3 is athero-protective	[33]
CHAER	*CHAER1/GM42105*	Mouse	Interacts with *PRC2*	Inhibition of Chaer expression in the heart before, but not after, the onset of pressure overload substantially attenuates cardiac hypertrophy and dysfunction	[34]
LIPCAR	*JA760602*	Human	-	Elevated in patients with chronic heart failure	[35]
MIAT	*MIAT/RNCR2*	Human	MIAT functioned as a ceRNA for miR-24 to modulate Furin and TGF-β1 expression	Involved in pathological angiogenesis and is suggested as a predictor of MI	[36]
MHRT	*MHRT*	Human	-	Protective factor for cardiomyocyte	[37]
GAS5	*GAS5*	Human, Mouse, Rat	Interacts with miR-290, Inhibits nuclear translocation of beta-catenin, inducing expression of downstream genes	GAS5 knockdown aggravate hypertension-induced microvascular dysfunction	[38]
MEG3	*MEG3*	Mouse	MEG3 directly binds with the p53 DNA binding domain	MEG3 is upregulated following ischemia and stroke	[39]
UCA1	*UCA1*	Human	Inhibit the expression of p27	Upregulated in the plasma of patients after MI	[40]
HIF1A-AS1	*HIF1A-AS1*	Human	-	Plays an important role in the pathogenesis of cardiovascular disease (CVD)	[41]
NPPA-AS1	*NPPA*	Human	Alternative splicing of the *NPPA* gene	Involved in CVD	[42]
CHRF	*DCC*	Human	Targeting miR-489	Regulates cardiac hypertrophy	[43]
CHAST	-	Mouse, Human	CHAST negatively regulated Pleckstrin homology domain–containing protein family M member 1	Potential target to prevent cardiac remodeling	[44]
PANCR	*PITX2*	Human	miR-143 and miR-501 sponge	Affected in atrial fibrillation (AF)	[45]
PVT1	*PVT1*	Mouse	Essential for the maintenance of cell size of cardiomyocytes	Regulation of cardiac hypertrophy	[46]
Carl	*CASC11*	Human	Targeting miR-539 and *PHB2*	Regulates mitochondrial fission and apoptosis in MI	[47]
HOTAIR	*HOTAIR*	Human	Targets expression of *NOX2*	Upregulated in ischemic heart failure	[48]

**Table 2:** List of clinically relevant miRNA in cardiovascular biology.

Transcript	Organism Studied	Mechanism	Disease	Reference
miR-133	Mouse, Human	Targets HAND-2, de-repression of *IRX5*	Regulates the balance between differentiation and proliferation during cardiogenesis	[52]
miR-208a	Mouse, Human	Regulates the balance between the a- and b-myosin heavy chains	MiR-208 inhibition is protective in heart failure	[53]
miR-17	Mouse	target genes are *BIM, BMP 2/4, ISL1, TBX1*	Regulation of cardiac progenitor genes, repression of fibronectin	[54]
miR-195	Human	*CHEK1* regulation	Associated with ventricular septal defect and right ventricular hypoplasia	[55]
miR-25	Mouse, Human	Regulation of cardiac contractility through control of SERCA2a expression	Mitigation of heart failure	[56]
miR-302/367	Mouse, Human	-	Role in differentiation and reprogramming in cardiac remodeling	[57]
miR-590	Mouse, Rat	-	Stimulates cardiac regeneration	[58]
miR-99a	Mouse, Zebrafish	Regulates *FNTB, SMARCA5* expression	Regulate cardiac regeneration	[59]
miR-499	Mouse, Human	Regulates *SOX6, ROD1, MYH7B*	Reduces cell proliferation and enhances myocyte differentiation	[60]
miR-15a/b	Mouse, Pigs	Targets *BCL2* and *ARL2* in cardiomyocytes	Reduced infarct size and cardiac remodeling and enhances cardiac function in response to myocardial infarction (MI)	[61]
miR-145	Mouse, Human	Regulates *KLF4* and *KLF5* expression	Prevented the development of pulmonary artery hypertension	[62]
miR-320a	Mouse, Rat	Reduced infarct size via antithetical regulation of heat-shock protein-20	Potential therapeutic target for ischemic heart disease	[63]
miR-22	Human, Mouse, Rat	Regulates *MYH7* expression	Deregulated in human heart failure and also in animal models of cardiac hypertrophy and failure	[64]
miR-21	Mouse	Regulates ER-MAP kinase activity	In vivo silencing of miR-21 in rodent model of cardiac fibrosis impacts global cardiac structure and ameliorates cardiac dysfunction	[51]

**Table 3:** List of clinically relevant circRNA in cardiovascular biology.

Transcript	Host Gene	Organism Studied	Mechanism	Disease	Reference
HRCR	*PWWP2A*	Mouse	miRNA sponge for miR-223	Inhibits hypertrophic cardiomyopathy and heart failure	[85]
MICRA	*ZNF609*	Human	-	Downregulated in heart failure	[86]
CDR1AS	*CDR1*	Mouse	miRNA sponge for miR-7	Upregulated in myocardial infarction	[87]
circFoxo3	*FOXO3*	Mouse	Retains ageing factors ID-1, E2F1, FAK, and HIF1α in cytoplasm	Upregulated in myocardial senescence	[88]
cZNF292	*ZNF292*	Human	-	Promotes angiogenesis	[89]
circANRIL	*CDKN2B-AS1*	Human	Binds to *PES1*	Protects against atherosclerosis	[90]

**Table 4:** Experimental techniques available for the identification, quantification, and characterization of ncRNAs.

Technique	Used for	Throughput
Microarrays	Quantification of transcript expression	High
Serial analysis of gene expression (SAGE)	Transcript identification and quantification of expression	High
Next generation sequencing (NGS)-based transcriptome analysis methods (RNA-seq—RNA sequencing, CAGE—Cap Analysis of Gene Expression, GRO-Seq—Genomic run-on sequencing, etc.)	Transcript identification and quantification of expression	High
Quantitative RT-PCR—Real time- polymerase chain reaction	Validation of transcript existence and abundance in real time	Low
RNA-Fluorescence in-situ hybridization (RNA-FISH)	Transcript Localization in the cellular compartment and relative abundance	Low
Northern Blot	transcript size, the observation of alternate splice products, the use of probes with partial homology, the quality and quantity of transcript	Low
RNA immunoprecipitation (RIP)	RNA–protein interaction	Moderate
Crosslinking immunoprecipitation sequencing (CLIP-Seq) based methods (HITS-CLIP—High-throughput sequencing, PAR-CLIP—photoactivatable ribonucleoside, iCLIP—individual-nucleotide resolution etc.)	RNA–Protein interaction	High
Chromatin isolation by RNA purification (ChIRP)	RNA–DNA interaction	High
DNA-RNA fluorescence in-situ hybridization (FISH)	RNA–DNA interaction	Low
Capture hybridization analysis of RNA targets (CHART)	RNA–DNA interaction (localization in the genome)	Moderate
RNA antisense purification (RAP)-DNA	RNA–DNA interaction	High
RNA antisense purification (RAP)-RNA	lncRNA-RNA interactions that occur through protein intermediates or through direct RNA-RNA hybridization	High
Cross-linking, ligation and sequencing of hybrids (CLASH)	RNA–RNA interaction	High
Clustered regularly interspaced short palindromic repeats (CRISPR) based techniques	Overexpression/Knockdown, interactions, cellular compartment localization of particular transcript	Low
Transcription activator-like effector nucleases (TALENs)	Knockout/overexpression of the transcript	Low
Zinc-finger nucleases (ZFNs)	Knockout/overexpression of the transcript	Low
Antisense oligos (ASOs), Locked nucleic acids (LNAs) based methods	Silencing of transcript	Low
RNA interference (RNAi)	Silencing of transcript	Low
Luciferase Reporter Assays	Target sites of the transcript	Low
RNase protection assays	Transcription start-site localization	Low

**Table 5:** Studies looking at lncRNA conservation.

Study	Species/Organisms	Details	Reference
Necsulea et al.	11 vertebrates	RNA sequencing (RNA-seq) of multiple tissues	[113]
Washietl et al.	6 mammals	RNA-seq of multiple tissues	[112]
PLAR	17 vertebrates	RNA-seq of multiple tissues	[114]
Lopez-Ezquerra et al.	7 insect species	Comparative analysis of long non-coding RNAs (lncRNAs) in insect species	[115]
Gardner et. al.	48 avian species	Comparative analysis of non-coding RNAs (ncRNAs) in avian genomes	[116]

**Table 6:** RNA secondary structure conservation-based studies.

Study	Technique/Tool Used	Reference
Washietl et al., 2005	RNAz	[127]
Pedersen et al., 2006	EvoFold	[128]
Washietl et al., 2007	AlifoldZ, RNAz, EvoFold	[129]
Torarinsson et al., 2008	CMfinder, RNAz, EvoFold	[130]
Rabani et al., 2009	RNApromo	[131]
Parker et al., 2011	EvoFam	[132]
Smith et al., 2013	RNAz and SISSIz	[119]
Will et al., 2013	RE-Alignment for Prediction of structural ncRNA (REAPR)	[133]
Seemann et al., 2017	CMfinder	[134]
Ding et al., 2014	Structure-seq	[135]
Rouskin et al., 2014	Dimethyl sulfate sequencing (DMS-seq)	[136]
Wan et al., 2014	Parallel Analysis of RNA Structure (PARS)	[137]
Aw et al., 2016	Sequencing of psoralen crosslinked, ligated, and selected hybrids (SPLASH)	[138]
